# Current Epidemiology of *Pneumocystis* Pneumonia

**DOI:** 10.3201/eid1010.030985

**Published:** 2004-10

**Authors:** Alison Morris, Jens D. Lundgren, Henry Masur, Peter D. Walzer, Debra L. Hanson, Toni Frederick, Laurence Huang, Charles B. Beard, Jonathan E. Kaplan

**Affiliations:** * University of Southern California, Los Angeles, California, USA;; † University of Pittsburgh School of Medicine, Pittsburgh, Pennsylvania, USA;; ‡ University of Copenhagen, Hvidovre, Denmark;; § National Institutes of Health, Bethesda, Maryland, USA;; ¶ University of Cincinnati, Cincinnati, Ohio, USA;; # Centers for Disease Control and Prevention, Atlanta, Georgia, USA;; ** University of California San Francisco, San Francisco, California, USA;; †† Centers for Disease Control and Prevention, Fort Collins, Colorado, USA

**Keywords:** Pneumocystis jirovecii, Pneumocystis pneumonia, PCP, epidemiology, HIV, highly active antiretroviral therapy, perspective

## Abstract

*Pneumocystis* pneumonia (PCP) has historically been one of the leading causes of disease among persons with AIDS. The introduction of highly active antiretroviral therapy in industrialized nations has brought about dramatic declines in the incidence of AIDS-associated complications, including PCP. In the adult population, the incidence of PCP has significantly decreased, but it remains among the most common AIDS-defining infections. Similar declines have been documented in the pediatric population. In much of the developing world, PCP remains a significant health problem, although its incidence among adults in sub-Saharan Africa has been debated. This review discusses the epidemiology of PCP during the current era of the AIDS epidemic. Although fewer cases of PCP occur in industrialized countries, increasing drug-resistant HIV infections, possible drug-resistant PCP, and the tremendous number of AIDS cases in developing countries make this disease of continued public health importance.

## Abstract 2

Changes in incidence of PCP, groups at risk for PCP, and possible trends in the disease are discussed.

*Pneumocystis* pneumonia (PCP), which is caused by *Pneumocystis jirovecii* (formerly *P. carinii* f. sp. *hominis*), is frequently the first serious illness encountered by HIV-infected persons. During the early years of the AIDS epidemic, PCP was the AIDS-defining illness for as many as two thirds of patients in the United States. Although a decline in incidence of PCP occurred during the era of highly active antiretroviral therapy (HAART), PCP remains the most common serious opportunistic illness in HIV-infected persons (*1*). Patients in the developing world without access to PCP prophylaxis or antiretroviral drugs remain at high risk, and PCP continues to develop in certain groups in industrialized countries.

The drug of choice for treatment and chemoprophylaxis of PCP is trimethoprim-sulfamethoxazole (TMP-SMX). In recent years, antimicrobial drug resistance has emerged as a possible cause of failure of patients to respond to TMP-SMX. Investigators have demonstrated an association between exposure to sulfa drugs and mutations in the dihydropteroate synthase (DHPS) gene of *P. jirovecii*, but the relationship between these mutations and treatment (or prophylaxis) failure is unclear. Understanding whether DHPS mutations cause antimicrobial drug resistance is important in guiding clinicians who care for patients with PCP.

A series of articles in this issue of Emerging Infectious Diseases highlights the continuing importance of PCP, the potential for drug resistance, and laboratory techniques that can be used to study the problem. We hope that these articles will stimulate interest in exploring the relationship between DHPS mutations and resistance of *P. jirovecii* to sulfa-containing drugs and in assessing DHPS mutations as possible causes of treatment failure in patients with PCP. In this introductory article, we summarize the changes in incidence of PCP since the introduction of HAART, discuss groups at risk for PCP in developing and industrialized nations, and examine possible future trends in the disease. A data collection form has been included online with this series of articles to assist in the collection of appropriate and standardized data from patients with PCP and to facilitate comparing and pooling data from different centers (Appendix).

## PCP before HAART

The first clinical cases of PCP were reported during World War II in orphanages in Europe. These cases of "plasma cell pneumonia" were common among malnourished children and were later reported in children in Iranian orphanages. The disease was then recognized in patients who were immunocompromised because of malignancies, immunosuppressive therapy, or congenital immunodeficiencies. Solid organ transplantation increased the number of patients at risk for PCP, although rates diminished after chemoprophylaxis was introduced. Without chemoprophylaxis, rates of PCP are 5%–25% in transplant patients, 2%–6% in patients with collagen vascular disease, and 1%–25% in patients with cancer. Defects in CD4+ lymphocytes are a primary risk factor for developing PCP, but the immune response to *Pneumocystis* is complex. CD8+ lymphocytes seem to be important in *Pneumocystis* clearance, and defects in B-cells and antibody production may also predispose to PCP.

The beginning of the AIDS epidemic in the early 1980s shifted the incidence of PCP from a rare disease to a more common pneumonia. Clusters of PCP cases in homosexual men and intravenous drug users were one of the first indications of the HIV epidemic (*2*). PCP rapidly became the leading AIDS-defining diagnosis in HIV-infected patients. In the initial stages of the epidemic, PCP rates were as high as 20 per 100 person-years for those with CD4+ cell counts <200 cells/µL (*3*). PCP was responsible for two thirds of AIDS-defining illnesses, and an estimated 75% of HIV-infected patients would develop PCP during their lifetime (*4*).

The first substantial decline in the incidence of PCP occurred after the introduction of anti-*Pneumocystis* prophylaxis in 1989 (*5*). Although absolute numbers of cases of PCP as an AIDS-defining illness in the United States remained stable from 1989 to 1992 because of an increasing incidence of AIDS, the percentage of AIDS cases with PCP declined from 53% in 1989 to 49%, 46%, and 42% in 1990, 1991, and 1992, respectively (Centers for Disease Control and Prevention, AIDS Surveillance Summaries, 1989–1992). The later use of combination antiretroviral therapy further reduced the rates of PCP among adults by 3.4% per year after 1992 (*1*).

## PCP in Adults in Industrialized Countries after HAART

### Incidence

**Figure 1:**
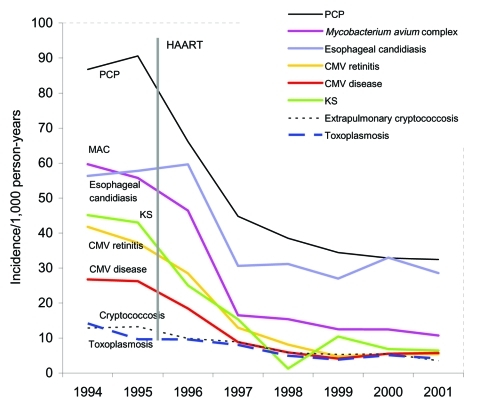
Yearly opportunistic infection rates per 1,000 person-years, CDC Adult and Adolescent Spectrum of Disease Project, 1994–2001. CMV, cytomegalovirus; HAART, highly active antiretroviral therapy; KS, Kaposi's sarcoma; MAC, *Mycobacterium avium* complex; PCP, *Pneumocystis* pneumonia. Data are standardized to the population of AIDS cases reported nationally in the same year by age, sex, race, HIV exposure mode, country of origin, and CD4+ lymphocyte count.

The advent of HAART has resulted in further declines in rates of PCP and other opportunistic infections (*1*). Several large, multicenter studies have specifically tracked the incidence and epidemiologic features of PCP. The largest is the Adult and Adolescent Spectrum of HIV Disease (ASD) Project. Data from this project indicated a marked reduction in the incidence of all opportunistic infections in 1996 and 1997, when HAART first became widely available (Figure 1). PCP cases decreased 3.4% per year from 1992 through 1995; the rate of decline of PCP increased to 21.5% per year from 1996 through 1998 (*1*). Despite this improvement, PCP is still the most common AIDS-defining opportunistic infection in the United States.

The Multicenter AIDS Cohort study (MACS) has followed >5,000 homosexual men since 1984 (*6*). Of these, 2,195 were either HIV-infected at time of enrollment or seroconverted to HIV during the study. Opportunistic infection rates were compared for the HAART era (1996–1998) and the era of antiretroviral monotherapy (1990–1992) (*7*). For persons who seroconverted during the study period, the relative hazard for development of PCP from seroconversion to initial AIDS-defining opportunistic infection was 0.06 during the HAART era compared to the time of monotherapy. For those already diagnosed with AIDS, the study found a hazard of 0.16, which demonstrated a dramatically lower risk for PCP during the HAART era.

In Europe, the EuroSIDA study has followed a cohort of >8,500 HIV-infected patients. The investigators examined changes in incidence of AIDS-defining illnesses before and after HAART was introduced and found results similar to those in North America (*8*). PCP cases decreased over time (1994–1998). Incidence of PCP fell from 4.9 cases per 100 person-years before March 1995 to 0.3 cases per 100 person-years after March 1998 (*9*).

### Occurrence in Relation to PCP Prophylaxis

**Figure 2:**
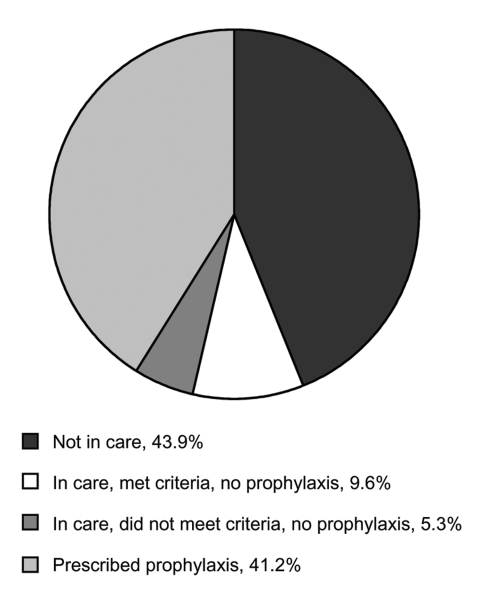
Classification of *Pneumocystis* pneumonia cases from 1999–2001, CDC Adult and Adolescent Spectrum of HIV Disease Project, n = 1,073.

PCP still occurs in industrialized nations despite the availability of HAART and anti-*Pneumocystis* prophylaxis. ASD investigated the history of prescriptions for PCP prophylaxis in HIV-infected adults in whom developed PCP from 1999 through 2001 (Figure 2). Almost 44% of PCP cases occurred in patients not receiving medical care, most of whom were probably not known to be HIV-infected. Forty-one percent of patients were prescribed prophylaxis but did not adhere to treatment, or PCP developed despite their taking medications appropriately. Possible explanations for PCP in the "breakthrough" group include the development of drug-resistant *Pneumocystis* or decreased efficacy of prophylaxis in those with low CD4+ cell counts. An additional 9.6% of patients were under medical care and should have received prophylaxis based on current recommendations, but had not been prescribed prophylaxis by their providers. Five percent of patients were under care but did not meet criteria for prophylaxis.

### Risk Factors

A CD4+ cell count <200 cells/µL was the leading pre-HAART risk factor for PCP and remains an important risk factor in the HAART era. The risk for PCP increases exponentially the lower the CD4+ cell count is below 200 cells/µL (*10*). When patients on HAART have sustained increases in CD4+ cell counts >200 cells/µL, the risk for PCP decreases sufficiently to safely discontinue both primary and secondary prophylaxis (*9**,**11*). Those in whom PCP develops while on HAART typically have low CD4+ cell levels. ASD found that the median CD4+ cell count in persons with PCP while on HAART was extremely low (29 cells/µL), although the count was somewhat higher than for those not on HAART (13 cells/µL) (*1*). The EuroSIDA study reported that persons on HAART in whom PCP developed had a median CD4+ cell count of 30 cells/µL, identical to those with PCP who were not receiving HAART (*8*). Patients without improvement in their CD4+ cell count despite use of HAART remain at risk for PCP, and PCP still rarely occurs in persons with CD4+ cell counts >200 cells/µL.

Other clinical factors such as sex, race or ethnicity, and HIV transmission category have been examined as risk factors for PCP. Men and women appear to have an equivalent risk for PCP (*12*). One study demonstrated that African Americans have approximately one third the risk for PCP as white persons (*10*), but this finding has not been replicated (*12*). PCP risk according to HIV transmission category is also debated. One autopsy study found that PCP was less common in intravenous drug users than in other risk groups (*13*). Kaplan et al. found a slightly increased risk for those men who had sex with men and were intravenous drug users, but risk was equivalent in other transmission categories (*12*).

### Risk for *Pneumocystis* Colonization

Although PCP cases have declined, polymerase chain reaction (PCR) has led to the discovery of *Pneumocystis* DNA in asymptomatic persons. *Pneumocystis* in respiratory specimens from persons who do not have signs or symptoms of clinical infection and who do not progress to infection has been defined as colonization or subclinical carriage. Often, *Pneumocystis* DNA is detected only by PCR, and the organism is not seen on routine histochemical staining. The clinical significance of *Pneumocystis* in respiratory specimens and the viability of organisms detected only by PCR are unknown. However, colonization may be important for several reasons. *Pneumocystis* colonization may increase the risk for progression to PCP, carriers of the organism may transmit infection to others, and latent infection may lead to inflammation that is detrimental to the lung. Most healthy persons do not have detectable *Pneumocystis* in respiratory specimens, but rates of colonization may be as high as 69% in HIV-infected persons (*14*). Recent evidence suggests that non–HIV-infected persons may also be colonized with *Pneumocystis*, thus increasing the potential number of persons affected (*15*).

## PCP in Children in Industrialized Countries

### Incidence

**Figure 3:**
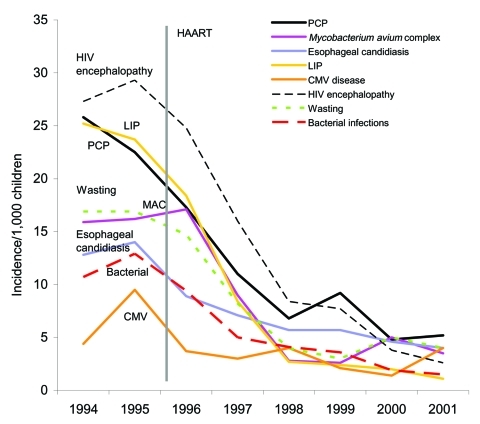
Yearly opportunistic infection rates per 1,000 HIV-infected children, CDC Pediatric Spectrum of Disease Project, 1994–2001. Bacterial, bacterial infections; CMV, cytomegalovirus; HAART, highly active antiretroviral therapy; LIP, lymphocytic interstitial pneumonia; MAC, *Mycobacterium avium* complex; PCP, *Pneumocystis* pneumonia. Incidence rates were calculated per 1,000 children at risk each year. All trends were significant at p < 0.05 in chi-square for trend analysis for four age groups (<1 year, 1–5 years, 6–9 years, and >10 years) except for the <1-year-old group for PCP, bacterial, and MAC.

Early in the HIV epidemic, PCP occurred in HIV-infected children at a rate of 1.3 cases per 100 child-years from infancy to adolescence and was as high as 9.5 cases per 100 child-years in the first year of life (*16**,**17*). In the 1990s, pediatric HIV infection decreased, primarily as a result of improved prenatal HIV testing and use of HIV treatment to prevent vertical transmission of the virus. The Pediatric Spectrum of Disease (PSD) study found significant decreases in the rates of most opportunistic infections in HIV-infected children during the HAART era (Figure 3). PCP cases declined significantly from 1992 to 1997, with an increase in the rate of decline after 1995, presumably from HAART (*1*). Because widespread use of HAART for children has occurred more recently than for adults, the full effect of HAART on pediatric PCP likely has not yet been realized.

### Risk Factors

The occurrence of PCP in infants does not seem to be related to the CD4+ cell count in the same manner as in adults, although it is related to the percentage of CD4+ cells and CD4+ cell counts are below normal in children <1 year of age with PCP (*18*). Furthermore, peak incidence of PCP occurs in infants 3–6 months of age, when HIV status may still be undetermined. Implementing recommendations to initiate PCP prophylaxis in all infants born to HIV-positive mothers decreased the incidence of the disease in the pediatric population before the advent of HAART (*11*). For children older than 6 years of age, the CD4+ cell count predicts disease in a manner similar to adults, and CD4+ cell counts <200 cells/µL are still considered an indication for prophylaxis (*11*).

Although HAART decreased the incidence of PCP in children, it has not eliminated the disease, mostly because of failure to identify HIV-infected mothers. PCP seems to occur early in life among HIV-infected infants, which suggests that exposure to *Pneumocystis* is common. In fact, anti-*Pneumocystis* antibodies develop in most nonimmunocompromised children in the first several years of life (*19**,**20*). A British study of children with PCP in the beginning of the HAART era found that PCP developed in 83 of 531 children with perinatally acquired HIV as their first AIDS indicator disease, which represented≈50% of AIDS diagnoses (*21*). Most of these children were <12 months of age, and 79% were born to mothers not previously diagnosed with HIV. Given that the mothers had unrecognized HIV disease, HAART would be expected to have little effect on disease incidence in this population, and improved maternal screening would be more important for disease prevention.

### Risk for *Pneumocystis* Colonization

Colonization may occur at higher rates in healthy children than in healthy adults. Vargas recently documented that nested PCR was positive for *Pneumocystis* DNA in the nasopharyngeal aspirates of 32% of 74 healthy Chilean infants (*20*). Children dying of sudden infant death syndrome (SIDS) also have a high rate of *Pneumocystis* (51 [30%] of 171), as seen on microscopy of lung specimens (*22*). The role *Pneumocystis* plays in SIDS is not understood. Similar to the adult population, effects of *Pneumocystis* colonization and relationship to PCP developing or transmission of infection are not known.

## PCP in the Developing World

In contrast to the dramatic improvements in the industrialized world, persons living in developing nations continue to be devastated by HIV. The World Health Organization estimates that 42 million persons were living with HIV at the end of 2002 and that 95% of these persons live in developing countries. Large portions of the populations of Southeast Asia and sub-Saharan Africa are infected with HIV, and an estimated 55 million persons will die of AIDS in sub-Saharan Africa from 2000 through 2020. HIV is also increasing in areas such as Latin America, eastern Europe, and Asia. Despite increasing efforts to supply affordable therapy to these nations, HAART is not widely available.

PCP still occurs frequently in many parts of the developing world (*23*). Studies from Thailand show a prevalence of 27% to 40% among HIV-infected patients treated at a university hospital clinic (*24**,**25*). Central and South America also have a large number of PCP cases. One Brazilian study found that 55% of HIV-infected persons with respiratory complaints were diagnosed with PCP, although a small autopsy study of hospitalized Brazilian patients found only 13% to have PCP (*26**,**27*). Other studies in this region report PCP prevalence from 24% to 29%, depending on the population studied (*28**,**29*).

## PCP in Adults in Africa

In contrast to the situation in many other developing regions, PCP has been thought to be rare in African adults. Several representative series are summarized in Table 1. Most early studies reported prevalence rates of 0% to 11% in HIV-infected patients (*30**,**31**,**33**,**34*), although one early study found a rate of 33% (*35*).

**Table 1:** Summary of selected studies of *Pneumocystis* pneumonia in African adults^a^

Study (ref)	Site	Study period	No. patients	Population	Diagnostic sample/method	% with PCP (n)
Abouya, 1992 (*30*)	Cote d'Ivoire	1989	53	Died while inpatient	Autopsy lung tissue/Grocott	8 (4)
Ansari, 2002 (*31*)	Botswana	1997–1998	104	Died while inpatient	Autopsy lung tissue/Grocott	11 (11)
Aderaye, 2003 (*32*)	Ethiopia	1996	119	Symptomatic, AFB negative, outpatients	Expectorated sputum/IF	11 (13)
Batungwanayo, 1994 (*33*)	Rwanda	1990	111	Symptomatic, AFB negative, outpatients	BAL, Tbbx/methenamine silver	5 (5)
Kamanfu, 1993 (*34*)	Burundi	1991	222	Symptomatic, hospitalized	BAL/Giemsa, IF	5 (11)
Malin, 1995 (*35*)	Zimbabwe	1992–1993	64	Symptomatic, hospitalized, AFB negative	BAL/methenamine silver, diff-quik, toluidine blue-O	33 (21)
Worodria, 2003 (*36*)	Uganda	1999–2000	83	Symptomatic, hospitalized, AFB negative	BAL/IF	39 (32)

^a^AFB, acid-fast bacilli; BAL, bronchoalveolar lavage; IF, immunofluorescence; Tbbx, transbroncial lung biopsy.

PCP might not have been commonly reported in Africa for several reasons. Limited resources for diagnosis may have led to lower estimates of PCP. Experienced laboratory personnel are required to prepare and interpret diagnostic specimens. Bronchoscopy is expensive, and induced sputum also requires specialized equipment and personnel to obtain adequate samples. Limited resources make empiric therapy of HIV-infected persons with pneumonia common, possibly leading to inaccurate estimates of the true incidence of PCP. HIV-infected African adults also have high rates of bacterial pneumonia and tuberculosis, diseases that may result in death at higher CD4+ cell counts and prevent many HIV-infected patients from reaching a stage at which they would be susceptible to PCP. Environmental factors, such as seasonal variations, might contribute to a low rate of PCP in Africa. However, high rates of anti-*Pneumocystis* antibodies among African children suggest that exposure to the organism is common (*19*). Regional strains may be less virulent, or the population may be more resistant, as HIV-infected African Americans have been shown to have lower PCP rates compared to white Americans (*10*). Detailed molecular study of the organism in different parts of the world is needed to resolve these issues.

The incidence of PCP in Africa may be growing as the AIDS epidemic progresses. A recent review concluded that cases of PCP seem to have increased over time (*23*), but whether this increase resulted from actual changes in PCP incidence or from improved detection techniques is unclear. Some studies have reported higher rates of PCP in Africa compared to past findings (Table 1). Malin et al. studied a group of 64 hospitalized HIV-infected patients in Zimbabwe in 1995 (*35*). These patients had pneumonia unresponsive to penicillin, and sputum samples were smear-negative for acid-fast bacilli (AFB). All patients underwent bronchoscopy with bronchoalveolar lavage (BAL). Twenty-one (33%) of these patients had PCP. Reasons for a higher rate of PCP among these patients included use of definitive diagnosis and probable selection bias by including only patients with severe pneumonia when other diagnoses, such as tuberculosis, had been excluded. Another study examined 83 patients hospitalized with respiratory symptoms (*36*). All patients had sputum cultures that were negative for AFB and underwent bronchoscopy with BAL for diagnosis. Thirty-two patients (38.6%) were diagnosed with PCP. Not all studies have found high rates of PCP. Aderaye et al. reported that of 119 outpatients with respiratory symptoms and negative AFB cultures, only 11% had PCP (*32*). Similarly, another recent study found PCP in 11% of patients who underwent autopsy after dying as an inpatient with respiratory symptoms (31). Future research will be needed to clarify the risk for PCP in Africa.

### PCP in Children in Africa

In contrast to adults, HIV-infected children in Africa have high rates of PCP. Autopsy series describe rates of PCP from 14% to 51.3%, depending on the age group studied (Table 2). Ikeogu et al. found that in Zimbabwe, 19 (15.5%) of 122 HIV-infected children who died <5 years of age had evidence of PCP at autopsy (*40*). All cases except one were in infants <6 months. Another autopsy study from the early 1990s found that PCP was present in 11 (31%) of 36 HIV-infected infants but was not found in 42 HIV-infected children >15 months (*41*). The largest autopsy series examined 180 HIV-infected children in Zambia (*38*). Twenty-nine percent of the children died of PCP, making PCP the third leading cause of death overall. Among children <6 months of age, PCP was the most common cause of pneumonia, detected in 51.3%. Six of 84 HIV-negative children had evidence of PCP at autopsy. The most recent autopsy series reported that 10 (28.6%) of 35 HIV-infected children had PCP (*37*).

**Table 2:** Summary of selected studies of *Pneumocystis* pneumonia in African children^a^

Study (ref)	Site	Study period	No. patients	Population/age	Diagnostic sample/method	% with PCP (n)
Ansari, 2003 (*37*)	Botswana	1997–1998	35	Died while inpatient/1–13	Autopsy lung tissue/Grocott	29 (10)
Chintu, 2002 (*38*)	Zambia	1997–2000	180	Died from respiratory disease/1 mo–16	Autopsy lung tissue/methenamine silver	29 (52)
Graham, 2000 (*39*)	Malawi	1996	93	Hospitalized for pneumonia/2 mo–5	NPA/IF	17 (16)
Ikeogu, 1997 (*40*)	Zimbabwe	1992–1993	122	Died on arrival to hospital/<5	Autopsy lung tissue/methenamine silver	16 (19)
Lucas, 2000 (*41*)	Côte d'Ivoire	1991–1992	78	HIV-positive undergoing autopsy, inpatient or outpatient/1 mo–12	Autopsy lung tissue/unknown	14 (11)
Madhi, 2002 (*42*)	South Africa	2000–2001	185 (231 episodes)	Hospitalized with severe pneumonia/1–38 mo	IS, NPA/IF	44 (101)
Ruffini, 2002 (*43*)	South Africa	1999	105	Hospitalized with severe pneumonia/2–24 mo	IS, NPA/IF	49 (51)
Zar, 2001 (*44*)	South Africa	1998	151	Hospitalized with pneumonia/3–16 mo	BAL, IS, NPA/methenamine silver, IF	10 (15)

^a^BAL, bronchoalveolar lavage; IF, immunofluorescence; IS, induced sputum; NPA, nasopharyngeal aspirate.

Because autopsy studies examine terminal disease, their assessment of disease prevalence might be biased. Several authors described prevalence of PCP among children in clinic or hospital settings to estimate disease frequency more accurately. Most studies reported rates higher than those in adults. Two authors found rates >40% among HIV-infected children hospitalized with pneumonia (*42**,**43*). Ruffini studied children from 2 to 24 months of age with pneumonia and found that 48.6% had PCP (*43*). Madhi found that in 231 episodes of pneumonia in HIV-infected children, 101 (43.7%) were due to PCP (*39*). PCP was most common in infants <6 months, although 35.7% of pneumonias in older children were also caused by PCP. Graham, in a smaller study of 16 cases of PCP in 93 children with HIV infection, also found that most cases of PCP occurred in infants (*42*). The study reporting the lowest frequency of PCP among children with pneumonia found 15 (9.9%) of 151 HIV-infected children to have PCP (*44*). Four non–HIV-infected children also had PCP. The authors speculated that the lower rate of PCP in their study may have been attributable to their inability to follow negative sputum examinations with bronchoscopy.

## The Future of PCP

The decline in PCP incidence in the industrialized world may be short-lived. Although current regimens are effective in treating HIV, as many as 19% of patients starting HAART will have a viral level >10,000 copies/mL after 48 weeks of treatment (*45*). In the EuroSIDA cohort, an increasing proportion of HIV-infected patients have been exposed to all classes of antiretrovirals, with 47% of their cohort exposed to nucleoside reverse transcriptase inhibitors, protease inhibitors, and non-nucleoside reverse transcriptase inhibitors by 2001 (*45*). Of those patients in the cohort with multidrug-resistant HIV who received salvage regimens, a new AIDS-defining opportunistic infection developed in 11%. Growing transmission of resistant HIV is also likely. If new drugs do not become available, the number of patients with resistant virus and opportunistic infections, including PCP, will continue to climb.

Not only is HIV developing resistance, but *Pneumocystis* may also develop resistance to standard prophylaxis and treatment regimens. Many researchers have reported mutations of *Pneumocystis* in response to use of sulfa- or sulfone-containing anti-*Pneumocystis* regimens. Whether these mutations increase the likelihood of prophylaxis or treatment failure is unclear and is reviewed in other papers in this series.

## Conclusion

Despite the declines in death and disease from HIV in the United States and western Europe, PCP remains an important disease and is unlikely to be eradicated. In industrialized nations, PCP still occurs in those not yet diagnosed with HIV or not in medical care, those not receiving PCP prophylaxis, and those not taking or not responding to HAART. Resistance in HIV and *Pneumocystis* may contribute to future increases in PCP incidence. In most developing nations, AIDS patients are at high risk for PCP. In sub-Saharan Africa, the effect of disease from PCP in infants and children is high and is probably greater in adults than previously recognized. Colonization rates among both HIV-infected and non–HIV-infected populations may also be substantial. Better understanding of the epidemiology and transmission of PCP, and improved efforts in prevention and treatment, are needed.

## Supplementary Material

Appendix PNEUMOCYSTIS CARINII: SURVEILLANCE FOR DRUG-RESISTANCE PCP CHART ABSTRACTION FORM.
