# Supplementary material for: Current Epidemiology of Pneumocystis Pneumonia
Source: Emerg Infect Dis. 2004 Oct;10(10):1713–20. doi: 10.3201/eid1010.030985 (PMC3323247; doi:10.3201/eid1010.030985)
Supplement: Appendix — PNEUMOCYSTIS CARINII: SURVEILLANCE FOR DRUG-RESISTANCE PCP CHART ABSTRACTION FORM. [file 03-0985-app.pdf]

PNEUMOCYSTIS CARINII: SURVEILLANCE FOR DRUG-RESISTANCE
PCP CHART ABSTRACTION FORM
June 24, 2004

Type of Chart Abstraction: Positive………………..1
Negative………………2

PCP study number: |____|____|-|____|____|____|____|-|___|___|

Data abstractor's ID: |____|____|

Hospital Name: ____

Date of chart review: |___|___|-|___|___|-|___|___|___|___| (MM/DD/YYYY)

Date of admission: |___|___|-|___|___|-|___|___|___|___| (MM/DD/YYYY)

Date of discharge: |___|___|-|___|___|-|___|___|___|___| (MM/DD/YYYY)

SECTION A: BACKGROUND INFORMATION

A1. Patient Date of Birth: |___|___|-|___|___|___|___| (MM/YYYY)

A2. Patient State of Birth: ________________________________ /___/___/

If outside of the U.S., country of birth ________________________________ /___/___/
(If unknown, code as / 9 / 9 /)

A3. State and Zip Code of Current Residence:

CITY ___________________
STATE ___________________
ZIP CODE ___________________

(For unknown Zip code, code as “99999")
(For homeless, code as “00000”)

A4. Patient Gender (at birth): Male..........................1
Female.......................2

A5a. What ethnic group is the subject considered to be?

- ❑ Hispanic/Latino
- ❑ Not Hispanic/Latino
- ❑ Not Specified

A5b. What racial group is the subject considered to be?
(Other is not an option. Respondents must be instructed that they may choose more than one option)

- ❑ White
- ❑ African American or Black
- ❑ Asian
- ❑ American Indian or Alaska Native
- ❑ Native Hawaiian or other Pacific Islander
- ❑ Not Specified

A6. Was patient aware of his/her HIV infection before this admission?

Yes…………………………………. 1
No………………………………….. 2
Unknown…………………………... 9

A7. Date of first laboratory diagnosis of HIV: |___|___|-|___|___|-|___|___|___|___|
MM-DD-YYYY

(Code with year only if month and day unknown. If HIV infection first documented during this hospitalization, enter date serum specimen for HIV test was drawn. If HIV infection has never been documented, leave blank.)

A8. Indicate the reliability of information on date of HIV infection (circle one).

Date documented in chart by previous lab report…………………. 1
Date mentioned in a clinic/admission note………………………... 2
Other………………………………………………………………….. 3

A9. Indicate if the patient had a previous CD4 count documented to be <200 or had an AIDS-defining illness.
Note to abstractors: some typical AIDS-defining illness: PCP, disseminated MAC, toxoplasmosis, microsporidiosis, thrush, CMV retinitis, chronic cryptosporidiosis, extrapulmonary TB.

Yes………………………………….. 1
No…………………………………… 2 (Go to A11)
Unknown…………………………… 9 (Go to A11)

A10. Enter earliest date that patient had an AIDS-defining illness or CD4 count < 200.

|___|___|-|___|___|-|___|___|___|___|
MM - DD - YYYY

A11. History of *Pneumocystis* pneumonia prior to this hospitalization?

Yes.. 1
No.. 2 (Go to next section)
Unknown.. 9 (Go to next section)

A12. Date of most recent PCP diagnosis |___|___|-|___|___|-|___|___|___|___|
(MM-DD-YYYY)

A13. This most recent PCP episode was (circle one):

Presumptive…….. 1
Lab diagnosed …… 2
Unknown………… 9

A14. Was treatment with TMP/SMX used during this most recent PCP episode?

Yes... 1
No... 2
Unknown................................. 9

SECTION X: DIAGNOSTIC EVALUATION

X1. Sputum Induction Result (PCP)

Positive..1
Negative..2
Not done..3 (Go to X3)

X2. Sputum Induction Date |___|___|-|___|___|-|___|___|___|___| (MM/DD/YYYY)

X3. Bronchoalveolar lavage

Positive..1
Negative..2
Not done..3 (Go to X5)

X4. Bronchoalveolar lavage Date |___|___|-|___|___|-|___|___|___|___| (MM/DD/YYYY)

X5a. Additional specimen

- ❑ Sputum (go to X5b)
- ❑ Bronchoalveolar lavage (go to X5c)
- ❑ Not done (go to X6)

X5b. Additional Sputum Result

Positive..1 (go to X5d)
Negative..2 (go to X5d)

X5c. Additional Bronchoalveolar Lavage Result

Positive..1 (go to X5e)
Negative..2 (go to X5e)

X5d. Additional Sputum Specimen Date |___|___|-|___|___|-|___|___|___|___|
(MM/DD/YYYY)

X5e. Additional Bronchoalveolar Lavage Specimen Date
|___|___|-|___|___|-|___|___|___|___|
(MM/DD/YYYY)

X6. If microscopic diagnosis of PCP made, indicate semi-quantitative result of FIRST positive specimen:

Rare..1
Few..2
Moderate..3
Many..4

X7. If NO microscopic diagnosis of PCP made, was the subject empirically treated for PCP?

Yes……………………………………………………1
No..2
Unknown...9

X8. List "other" concurrent AND contributory PULMONARY diagnosis(es) during this hospitalization:

________________________ ________________________ ________________________

________________________ ________________________ ________________________

________________________ ________________________ ________________________

SECTION B: LAB VALUES

Fill in the following table with the most recent CD4 count and viral load obtained before admission and the most recent values obtained during or after admission

B1.

	CD4	Date (MM - DD - YYYY)	
Before admission	___________	|___|___|-|___|___|-|___|___|___|___|	|____|
During/after admission	___________	|___|___|-|___|___|-|___|___|___|___|	|____|

B2.

	Viral Load (HIV RNA)	Date (MM - DD - YYYY)	
Before admission	___________	|___|___|-|___|___|-|___|___|___|___|	|____|
During/after admission	___________	|___|___|-|___|___|-|___|___|___|___|	|____|

B3. Indicate type of test used to determine viral load (circle appropriate test).

Quantitative PCR....................1
Branch Chain DNA.................2
NASBA...................................3
Unknown.................................4

B4. Please refer to SECTION X. Skip to B5.

B5. Enter first serum LDH value that best represents the level when patient was diagnosed with PCP. Use the following guideline for selecting the best date. If the patient had at least one LDH value obtained in the 14 days before the diagnosis of PCP, enter the value for the specimen closest to the date of diagnosis. If no LDH was obtained in the 14 days before the date of PCP diagnosis, but an LDH level was obtained within 7 days after the PCP diagnosis, enter the first LDH value obtained after PCP diagnosis. If no LDH value was obtained in either of these two windows, code as "00".

LDH value	Date collected (MM – DD - YYYY)
_______	|___|___|-|___|___|-|___|___|___|___|

B6. Enter first serum albumin value obtained near PCP diagnosis date – use same criteria as for LDH.

Albumin Date collected (MM – DD - YYYY)

_______ |___|___|-|___|___|-|___|___|___|___|

B6b. Patient weight after admission: ___________________ Circle one: Pounds Kilograms
Note: Record first or closest weight measured after admission.

|___|___|-|___|___|-|___|___|___|___| (MM-DD-YYYY)

B7. Indicate amount of oxygen that was ordered for the patient when they were admitted to their hospital room (not in ER).

Room air (no oxygen) …………………………. 1
2-6 liters of oxygen by nasal canula……………2
Oxygen by face mask ………………………….. 3
Patient was intubated ………………………….. 4
Other…………………………………………… 5

Specify:____
Unknown ………………………………………. 9

B8. Was an arterial blood gas (ABG) obtained within 24 hours before or after admission?

Yes.. 1
No... 2 (Go to B13)
Unknown................................. 9 (Go to B13)

B9. Date of ABG: |___|___|-|___|___|-|___|___|___|___|
(MM - DD - YYYY)

B10. If an ABG was obtained within 24 hours, enter the results of that ABG. If no room air ABG was obtained, enter the results of first ABG.

pH ________
pCO2 ________
pO2 ________

B11. Indicate whether or not ABG was obtained on room air and, if patient was receiving supplemental oxygen, how much:

Room air (no supplemental oxygen)............... 1 (Go to B13)
2-6 liters of oxygen by nasal canula................ 2 (Go to B13)
Oxygen by face mask ....….... 3 (Go to B13)
Patient was intubated ....…… 4 (Go to B12)
Unknown ....…….….. 9

B12. Deleted. Skip to B13.

B13. Was patient intubated at any time during this hospitalization?
Yes...1
No...2 (Go to B15)
Unknown..9 (Go to B15)

B14. First intubated on |___|___|-|___|___|-|___|___|___|___| (MM/DD/YYYY)

Last extubated on |___|___|-|___|___|-|___|___|___|___| (MM/DD/YYYY)

B15. Deleted. Skip to B16a.

B16a. Did patient have a pneumothorax at *admission*?
Yes... 1
No.. 2
Unknown... 9

B16b. Did patient develop a pneumothorax at *any time during* this hospitalization?
Yes... 1
No.. 2
Unknown... 9

B17. Date of entry to ICU: |___|___|-|___|___|-|___|___|___|___| (MM-DD-YYYY)

Number of days in ICU: |___|___|___|

SECTION C: PREHOSPITALIZATION MEDICATION HISTORY

In this section, record information about medications that the patient had been prescribed and should have been taking anytime in the 3 months before being admitted to the hospital with PCP.

C1. Was patient prescribed any medication as prophylaxis for PCP?
- Yes.. 1
- No... 2 (Go to C3)
- Unknown................................. 9 (Go to C3)

C2. Check all that were prescribed and indicate dose/frequency.

**PROPHYLAXIS**	**DOSE**	**Do chart notes indicate that patient might not have been taking prophylaxis prescribed? If yes, circle 'not taking as prescribed'**
Trimethoprim-Sulfamethoxazole (Bactrim/Septra/Sulfatrim)		Not taking as prescribed
Pentamidine (NebuPent/Pentam-300)		Not taking as prescribed
Dapsone		Not taking as prescribed
Atovaquone (Mepron)		Not taking as prescribed
Other (Specify below)		Not taking as prescribed
Specify Name		______________________<br>______________________<br>______________________

C3. Was patient prescribed any anti-retroviral medications?
- Yes.. 1 (Continue to C4)
- No... 2 (Go to Section D)
- Unknown................................. 9 (Go to Section D)

C4.

ANTI-RETROVIRALS	DOSE	Does chart note indicate that patient might not be taking anti-retrovirals prescribed? If yes, circle 'not taking as prescribed'
AZT/ZDV/Zidovudine (Retrovir)		Not taking as prescribed
3TC/Lamivudine (Epivir)		Not taking as prescribed
AZT+3TC combo (Combivir)		Not taking as prescribed
ddI/Didanosine (Videx)		Not taking as prescribed
ddC/Zalcitabine (HIVID)		Not taking as prescribed
d4T/Stavudine (Zerit)		Not taking as prescribed
ABC/Abacavir (Ziagen)		Not taking as prescribed
Saquinavir (Invirase/Fortovase		Not taking as prescribed
Ritonavir (Norvir)		Not taking as prescribed
Indinavir (Crixivan)		Not taking as prescribed
Nelfinavir (Viracept)		Not taking as prescribed
Amprenavir (Agenerase)		Not taking as prescribed
Tenofovir (Viread)		Not taking as prescribed
Nevirapine (Viramune)		Not taking as prescribed
Delavirdine (Rescriptor)		Not taking as prescribed
Efavirenz (Sustiva)		Not taking as prescribed
Lopinavir/Ritonavir (Kaletra)		Not taking as prescribed
AZT+3TC+ABC (Trizivir)		Not taking as prescribed
Other		Not taking as prescribed

SECTION D: PCP MEDICATIONS USED DURING HOSPITALIZATION

Do not include steroids or drugs used for PCP prophylaxis (e.g., Bactrim 1 pill/day or less; dapsone 100 mg/day by itself)

Do include: TMP-SMZ IV; TMP-SMZ po if dose >1 pill/day; pentamidine; clindamycin+primaquine; dapsone+trimethoprim; atovaquone 750 mg q8h)

**Drug**	**IV or PO**	**Dose**	**Frequency**	**Start Date (MM/DD)**	**Stop Date (MM/DD)**	**Improvement?**<br>1: Improved or continued to do well<br>2: Worsened or continued to be ill<br>**3: Unknown**	**Reason for changing/stopping medication:**<br>**1: Dose changed (drug stayed the same)**<br>**2: Improved, so changed to oral meds**<br>**3: Discharged (doing well)**<br>**4: Adverse reaction (changed to another drug)**<br>**5: Failed to improve (changed to another drug)**<br>**6: Died**<br>**7: Other (describe)**	**Number of Doses NOT Given as Prescribed: FIRST 7 DAYS of Tx**

**Drug**	**IV or PO**	**Dose**	**Frequency**	**Start Date (MM/DD)**	**Stop Date (MM/DD)**	**Improvement?**<br>1: Improved or continued to do well<br>2: Worsened or continued to be ill<br>**3: Unknown**	**Reason for changing/stopping medication:**<br>**1: Dose changed (drug stayed the same)**<br>**2: Improved, so changed to oral meds**<br>**3: Discharged (doing well)**<br>**4: Adverse reaction (changed to another drug)**<br>**5: Failed to improve (changed to another drug)**<br>**6: Died**<br>**7: Other (describe)**	**Number of Doses NOT Given as Prescribed: FIRST 7 DAYS of Tx**

D2. Was patient prescribed steroids (prednisone, prednisolone, solumedrol) as adjunct therapy for PCP?

Yes.. 1
No.. 2
Unknown.. 9

D3. Was the patients treated with TMP/SMZ (e.g. Bactrim, Septra) at any time during hospitalization?

Yes.. 1
No.. 2 (Go to Section E)

D4. If yes, was treatment changed to another drug during their hospitalization?

Yes.. 1
No.. 2 (Go to Section E)

D5. If yes, was treatment changed due to poor response?

Yes.. 1
No.. 2 (Go to Section E)
Unknown.. 9 (Go to Section E)

D6. How was poor response to therapy determined?
(Circle all that apply, then go to Section E)

Worsening CXR or failure to improve as noted in physician's notes……….1
Increasing or non-improving oxygen requirements………………………...2
Poor clinical response……………………………………………………3
Specify____ |___|___|
Other……………………………………………………………………..7
Specify____ |___|___|
Unknown…………………………………………………………………....9

SECTION E: PCP HOSPITALIZATION and POSTHOSPITALIZATION COURSE

E1. Did the patient die during this hospitalization?

Yes.. 1 (Go to E5)
No... 2

E2. Was patient rehospitalized for PCP within 1 month of discharge?

Yes.. 1
No..2 (Go to E4)
Unknown...9 (Go to E4)

E3. Date of rehospitalization: |___|___|-|___|___|-|___|___|___|___|
(MM - DD - YYYY)

E4. Did the patient die after discharge but within 6 weeks after discharge?

Yes.. 1
No... 2 (STOP)
Unknown.. 9 (STOP)

E5. Date of death: |___|___|-|___|___|-|___|___|___|___|
(MM - DD - YYYY)

E6. Primary cause of death?

PCP.. 1
Other respiratory cause of disease……. 2
Cardiac or cardiopulmonary death…… 3
Other.. 7
[Specify] ___________________________
____
____
Unknown.. 9
